# Metabolic Drivers of Invasion in Glioblastoma

**DOI:** 10.3389/fcell.2021.683276

**Published:** 2021-07-01

**Authors:** Joseph H. Garcia, Saket Jain, Manish K. Aghi

**Affiliations:** Department of Neurological Surgery, University of California, San Francisco, San Francisco, CA, United States

**Keywords:** glioblastoma, invasion, brain tumor, metabolism, microenvironment

## Abstract

Glioblastoma is a primary malignant brain tumor with a median survival under 2 years. The poor prognosis glioblastoma caries is largely due to cellular invasion, which enables escape from resection, and drives inevitable recurrence. While most studies to date have focused on pathways that enhance the invasiveness of tumor cells in the brain microenvironment as the primary driving forces behind GBM’s ability to invade adjacent tissues, more recent studies have identified a role for adaptations in cellular metabolism in GBM invasion. Metabolic reprogramming allows invasive cells to generate the energy necessary for colonizing surrounding brain tissue and adapt to new microenvironments with unique nutrient and oxygen availability. Historically, enhanced glycolysis, even in the presence of oxygen (the Warburg effect) has dominated glioblastoma research with respect to tumor metabolism. More recent global profiling experiments, however, have identified roles for lipid, amino acid, and nucleotide metabolism in tumor growth and invasion. A thorough understanding of the metabolic traits that define invasive GBM cells may provide novel therapeutic targets for this devastating disease. In this review, we focus on metabolic alterations that have been characterized in glioblastoma, the dynamic nature of tumor metabolism and how it is shaped by interaction with the brain microenvironment, and how metabolic reprogramming generates vulnerabilities that may be ripe for exploitation.

## Introduction

Glioblastoma (GBM) is a primary malignant brain tumor with a median survival under 2 years (Wen and Kesari, 2008; Stupp et al., 2019). The poor prognosis GBM caries is largely due to cellular invasion, which enables escape from resection and drives inevitable recurrence (de Gooijer et al., 2018; Wolf et al., 2019). To successfully invade, GBM cells must be motile and penetrate adjacent brain tissue (de Gooijer et al., 2018). Though it is largely agreed upon that specific molecular factors are the primary drivers of invasion, attempts to identify and target these drivers have been unsuccessful thus far (de Gooijer et al., 2018).

Clinical observations and experimental studies have led to numerous hypotheses attempting to define the factors which drive tumor invasion, but whether these changes are causative is widely contested (Hatoum et al., 2019; Vollmann-Zwerenz et al., 2020). Regardless, whether the sequential acquisition of random mutations in GBM might provide and select for invasive traits in individual cells, or the invasive predisposition of a tumor is already imprinted in the majority of cancer cells, invading cells need to be able to continuously adapt to changing environments during the invasive process (de Gooijer et al., 2018).

Subsequent studies have revealed differentially expressed genes at individual steps in the process of cellular invasion (Paw et al., 2015). The expression of gene clusters associated with rapid growth and proliferation have been proven to confer a selective advantage to cells invading into the adjacent brain microenvironment (Cuddapah et al., 2014). Invasion is facilitated by multiple factors including tumor-intrinsic factors, central nervous system-specific niches and the interaction between tumor cells and the cerebral microenvironment (Paw et al., 2015; de Gooijer et al., 2018). Furthermore, through the secretion of specific factors and extracellular vesicles, tumors can degrade structural matrices and avoid immune detection (Goldbrunner et al., 1998; Cuddapah et al., 2014).

More recently, attention has been given to the notion that invasive cells also require specific metabolic traits to survive and grow in new environments which substantially vary in their nutrient and oxygen availability from the tumor core (Marin-Valencia et al., 2012; Strickland and Stoll, 2017). Such metabolic rewiring can be controlled transcriptionally (i.e., epigenetic alterations), but also post-translationally or through metabolite availability (Strickland and Stoll, 2017). In this Review, we discuss the pathways implicated in glioblastoma’s metabolism and how they potentially contribute to invasive cells, the growing evidence for the dynamic metabolic shifts these cells display to survive and propagate during the different steps of the invasive transition, and how targeting metabolism can potentially be used to inhibit invasion in glioblastoma.

## Metabolic Pathways in Glioblastoma

### Carbohydrate Metabolism

Cellular growth and division depend on the uptake of nutrients from the surrounding environment, which are in turn metabolized to produce energy and maintain cellular homeostasis (Phan et al., 2014). The brain is highly metabolic and utilizes approximately 20% of the body’s total oxygen consumption as well as 60% of the body’s daily glucose intake (Mergenthaler et al., 2013). Additionally, the brain requires a constant supply of glucose because it lacks the ability to store glucose as glycogen. This high metabolic demand and the inability of the brain to store glucose effectively lead to glucose being the most widely available nutrient in the brain microenvironment (Thorens, 2012; Mergenthaler et al., 2013).

High glucose levels have been linked to increased tumor invasion and poor patient survival (Labak et al., 2016; Martin-McGill et al., 2018). To gain insight into the mechanistic pathways that link high glucose levels and GBM invasion, studies in other cancer types point to reasonable hypotheses. A study in Lung adenocarcinoma cells revealed high glucose levels induced increased heme oxygenase-1 (HO-1), and subsequent increases in tumor cell invasiveness via increased PI3K/akt signaling (Han et al., 2013; Sferrazzo et al., 2020). Moreover, silencing of HO-1 led to an attenuation of high glucose induced invasion. The expression of HO-1 is increased in GBM in comparison to both normal brain tissue and compared to lower grade tumors (Sferrazzo et al., 2020). While a promising lead, future studies will determine whether glucose drives invasion in GBM through direct mechanistic effects, or subsequent effects of its downstream metabolites.

In neurons and glia alike, glucose is metabolized via glycolysis into pyruvate, which then enter the tricarboxylic acid (TCA) cycle to generate ATP through oxidative phosphorylation (OXPHOS) (Thorens, 2012). In contrast to non-cancerous glial cells, GBM cells preferentially metabolize glucose into lactate despite the presence of ample oxygen (known as the “Warburg” effect) (Strickland and Stoll, 2017; Pirmoradi et al., 2019). It is hypothesized that this metabolic shift enables tumor cells to use glucose-derived carbons for the synthesis of essential cellular ingredients while generating sufficient ATP to fuel cellular reactions (Phan et al., 2014; Kalyanaraman, 2017).

Aside from the benefits glycolysis brings to invading tumor cells by providing carbon skeletons for cellular building blocks several glycolytic enzymes play a more direct role in invasion. Phosphoglucose isomerase (PGI) is a cytosolic enzyme that catalyzes the conversion of glucose-6-phosphate into fructose-6-phosphate in the second step of glycolysis (Zhu et al., 2014). PGI is a secreted protein that behaves as a potent cytokine in extracellular environment. It has been hypothesized that PGI is an autocrine motility factor (AMF), and a tumor-secreted cytokine that stimulates cell migration *in vitro* and metastasis *in vivo* (Zhu et al., 2014; Kathagen-Buhmann et al., 2016). Fructose-1,6-bisphosphatase (FBP1), a gluconeogenesis enzyme, which catalyzes the splitting of fructose-1,6-bisphosphate (F-1,6-BP) into fructose 6-phosphate, also plays an important role in the epithelial to mesenchymal transition EMT (Han et al., 2013; Shi et al., 2017). Loss of FBP1 in breast cancer cells induces EMT and increases invasiveness, the enzyme may perform a similar role in GBM (Shi et al., 2017). Pyruvate kinase (PK) mediates the final rate-limiting step of glycolysis by catalyzing the dephosphorylation of phosphoenolpyruvate (PEP) to pyruvate (Mukherjee et al., 2013). In colon adenocarcinoma cells, a PK subtype knockdown suppressed invasion through reduced EGFR signaling, perhaps shedding light on this glycolytic enzyme’s invasive role across cancer types (Han et al., 2013).

The Warburg effect is well studied in glioblastoma (Strickland and Stoll, 2017). Like many other aggressive tumors, GBM cells ultimately gain oncogenic signaling pathways that regulate cell survival, cell proliferation, and aerobic glycolysis (Kalyanaraman, 2017). This metabolic adaptation, while a promising target for genetic and pharmacologic interventions, is only scratching the surface concerning metabolism in GBM invasion.

When metabolizing glucose, invading tumor cells must also divert carbon from glycolysis into the pentose phosphate pathway (PPP) for nucleotide synthesis and to combat oxidative stress (Jin and Zhou, 2019). The PPP has two primary roles: the generation of reducing equivalents (oxidative phase), and the production of ribose 5-phosphate for nucleotide generation (non-oxidative phase) (Loreck et al., 1987).

The oxidative arm of the PPP utilizes G6P as its substrate and leads to the generation of NADPH. The non-oxidative reactions of the PPP lead to the generation of ribose-5-phosphate (R5P) for nucleotide biosynthesis (Loreck et al., 1987; Jin and Zhou, 2019). Many glycolytic enzymes including phosphofructokinase 1, phosphoglycerate mutase, and pyruvate kinase M2 (PKM2) are tightly controlled by tumor cells (Kowalik et al., 2017). Regulation of these glycolytic enzymes can result in accumulation of substrates leading into diversion of carbon toward R5P for nucleotide synthesis (Strickland and Stoll, 2017; Jin and Zhou, 2019). The non-oxidative arm of PPP is also important for tumor cells, based on higher expression and activity of transketolase, which correlates with the rate of tumor growth in some cancers, including GBMs (Jiang et al., 2014; Jin and Zhou, 2019). A further level of regulation for R5P synthesis is the ratio of NADP + /NADPH in cells through the oxidative arm of PPP. The reversible reduction of glucose-6-phosphase (G6P) by G6P dehydrogenase is associated with reduction of NADP to NADPH, a critical reducing agent for several reactions including fatty acid and glutathione synthesis, for building biomass and controlling oxidative stress (Loreck et al., 1987; Kathagen-Buhmann et al., 2016).

To meet GBM’s high metabolic demands, glucose uptake is increased through the upregulation of glucose transport proteins (Strickland and Stoll, 2017). Expression of GLUT1, and to a lesser extent GLUT3 and GLUT4, have been shown to be increased in GBM cells both in conjunction with the relative glucose concentrations in the tumor microenvironment (Labak et al., 2016). Additionally, signaling pathways upstream of the metabolic shifts seen at the tumor invasive edge have been tied to increased glucose transporter expression (Labak et al., 2016; Strickland and Stoll, 2017). These results in GBM as well as other cancer types suggest that GLUT proteins not only play a role as glucose transporters, but also acts as a regulator of signaling cascades in the invasive phenotype of GBM (Labak et al., 2016).

### Lipid Metabolism

Lipids are a diverse group of water-insoluble molecules essential for numerous biological processes (Snaebjornsson et al., 2020). Their functions at the cellular level include energy storage and homeostasis, maintaining structural integrity as components of cellular membranes, and acting as messengers in cellular signaling. There is increasing evidence that cancer cells develop specific alterations in several aspects of lipid metabolism to supply their high bioenergetic demand (Guo et al., 2013; Shakya et al., 2021). By reprogramming lipid metabolism tumor cells can effectively enhance processes central to invasion, including cell growth, proliferation, and motility (Guo et al., 2013; Shakya et al., 2021). Lipids are abundant in the cerebral microenvironment and play a fundamental role in normal astrocyte function (van Deijk et al., 2017; Barber and Raben, 2019). Given this evidence, lipid metabolism has become an attractive target for investigation in metabolic drivers of glioblastoma invasion.

Among lipids in the brain cholesterol is among the highest in its relative abundance and represents 20–25% of total body cholesterol (Pirmoradi et al., 2019). Due to the inability of peripheral cholesterol to cross the blood–brain barrier (BBB), the majority of cholesterol in the brain is generated via *de novo* biosynthesis by astrocytes and delivered to neurons within high-density lipoproteins containing apolipoprotein E (Apo-E) (Barber and Raben, 2019; Pirmoradi et al., 2019). Unlike benign non-cancerous astrocytes (Ahmad et al., 2019; Pirmoradi et al., 2019), the metabolic needs of GBM cells are supplied mainly by exogenously rather than endogenously synthesized cholesterol, and cholesterol uptake is a crucial step for growth and survival for GBM cells (Geng et al., 2016). Studies have indicated that this uptake largely is driven via upregulation of sterol regulatory element-binding protein (SREBP-1) (Geng et al., 2016).

In addition to increased extracellular cholesterol uptake, GBM cells exhibit dysregulated cholesterol efflux and synthesis (An and Weiss, 2016; Ahmad et al., 2019). At high cell density, benign astrocytes reduce intracellular cholesterol by both upregulating the cholesterol efflux transporter ABCA1, and by reducing expression of genes in the mevalonate pathway (Patel et al., 2019). Glioblastoma cells conversely do not display this density-dependent regulation and maintain cholesterol synthesis (Ahmad et al., 2019; Patel et al., 2019). This dysregulation combined with the enhanced cholesterol uptake exhibited by GBM cells leads to the increased cholesterol required by these invasive cells (An and Weiss, 2016).

Lipidomics studies have shown the presence of triglycerides in two biopsies from glioblastomas (GBM) that had no treatment, but absent in healthy adult brain. Given that triglycerides contain three fatty acids and act as energy storage in addition to unsaturated fatty acids prominent in high-grade intracranial tumors, it appears that gliomas have developed an altered metabolism for fatty acids (Guo et al., 2013). Triglyceride accumulation has been linked directly to invasion in prostate cancer, and it is possible a similar mechanism occurs in GBM (Schlaepfer et al., 2015). Given these high levels of polyunsaturated fatty acids are present in gliomas, it is worth investigating whether the conversion of saturated fatty acids to unsaturated acids is a targetable driver of tumor invasion.

Fatty acid (FA) synthesis and catabolism are also among the lipid metabolic pathways with demonstrated alteration in Glioblastoma, with paradoxical elevation observed in both pathways. Fatty acids are able to cross the BBB and are readily available at the tumor’s invasive front (Barber and Raben, 2019; Taïb et al., 2019). The Fatty acid synthesis genes ACC and FAS have both been demonstrated to be highly expressed in glioblastoma and have been associated with poor patient outcomes (Zhao et al., 2006; Taïb et al., 2019). Genetic inhibition of both genes has been demonstrated to significantly suppress tumor growth *in vitro* and in xenograft mouse models (Guo et al., 2013; Yasumoto et al., 2016). Global metabolic profiling has suggested fatty acid oxidation is also upregulated in high grade glioma cells (Taïb et al., 2019). The simultaneous upregulation of these pathways could potentially supply GBM cells with ample lipids for cellular components while also allowing metabolic flexibility to invasive cells encountering novel microenvironments.

On a mechanistic level, expression of the FA uptake channel CD36, is upregulated under hypoxia in several types of cancer (Zhao et al., 2006; Liang et al., 2018, 36). CD36 has been previously implicated in tumor invasion and progression. Metastasis-initiating cells in human oral carcinomas display high levels of the CD36 (Liang et al., 2018, 3). Clinical data implies that the presence of CD36 + cells correlates with a poor prognosis and greater invasion numerous types of carcinomas, and inhibition of CD36 also impairs metastasis, in human melanoma and breast cancer-derived tumors (Ben-Shoshan et al., 2008; Liang et al., 2018, 3). In GBM, CD36 is expressed in tumorigenic cancer stem cells, and is associated with a pro-invasion phenotype (Hale et al., 2014). Further studies are required to study mechanism by which this channel protein drives invasion.

### Amino Acid Metabolism

Amino acids are increasingly recognized as important fuels for supporting cancer growth and division (Lieu et al., 2020). Biosynthetic and bioenergetic pathways alike rely on various amino acid contributors (Wu, 2009). Additionally, the breakdown of amino acids produces derivatives that can support tumor growth and invasive potential (Panosyan et al., 2017; Lieu et al., 2020). The diversity of roles amino acid metabolism plays in bioenergetic regulation, the synthesis of essential biomolecules, and in homeostatic maintenance, have made amino acid metabolism increasingly popular in the research of many cancer types including glioblastoma (Reeds et al., 1998; Bianchi et al., 2004; Wu, 2009).

Among the most intriguing amino acids in the study of glioblastoma invasion is glutamine (Obara-Michlewska and Szeliga, 2020). Glutamine is abundant in the cerebral microenvironment and serves as the primary precursor to the excitatory neurotransmitter glutamate (Smith, 1990; Tardito et al., 2015). Astrocytes play a key role in maintaining glutamine homeostasis in the brain by regulating its synthesis via glutamate recycling (Smith, 1990; Schousboe et al., 2014). Following this conversion, glutamine is released into the extracellular space through N or sodium-coupled amino acid transporters (SNATs) (Natarajan and Venneti, 2019). Gliomas take in the recycled glutamine through the upregulation of glutamine and glutamate transporters (Obara-Michlewska and Szeliga, 2020). Gene expression profiling has shown an upregulation of the glutamine importer ASCT2 (SLC1A5) compared to low grade gliomas, and glutamine deprivation has slowed GBM tumor growth in some *in vitro* studies (Hassanein et al., 2013; Obara-Michlewska and Szeliga, 2020).

There are a variety of potential roles played by glutamine as a driver of invasion in glioblastoma. Glutamine is the obligate nitrogen donor in several enzymatic reactions in the formation of both purines and pyrimidines (Natarajan and Venneti, 2019). The nucleotides formed by these reactions are essential to the growth and division required for tumor invasion. Glutamine also contributes to the pool of available non-essential amino acids (NEAAs) mitochondrial substrates in rapidly proliferating tumor cells (Obara-Michlewska and Szeliga, 2020). Finally, glutamine has been shown to stimulate mechanistic target of rapamycin (mTOR) complex 1 (mTORC1) signaling via translocation to the cellular lysosome. This pathway has been indicated as a potent driver of GBM growth and progression (Jewell et al., 2015; Natarajan and Venneti, 2019).

Glutamine starvation has also been recently been associated with tumor invasion, through the amino acid’s association with Cancer Associated Fibroblasts (CAFs), which facilitate epithelial tumor cell invasion (Schousboe et al., 2014; Mestre-Farrera et al., 2021). CAFs and other mesenchymal cells appear to rely on glutamine metabolism to a much greater degree than their epithelial counterparts. Deprivation of glutamine caused CAF and subsequent tumor invasion toward glutamine sources (Mestre-Farrera et al., 2021). Given the importance of CAFs in glioblastoma’s invasive capacity, it may be worthwhile to investigate the role of glutamine seeking behavior as a driver of tumor invasion.

In addition to glutamine, several other amino acids are utilized to fuel bioenergetic reactions and the synthesis of macromolecules in GBM (Panosyan et al., 2017; Lieu et al., 2020). Arginine has demonstrated importance in GBM cell adhesion, and it is been speculated that the molecular mechanisms that drive this process are also important for tumor cell migration and invasion (Pavlyk et al., 2015; Agarwal et al., 2017). Aspartate has been shown to be a limiting metabolite for glioblastoma cellular proliferation in hypoxic conditions, which has important implications as these tumors typically outgrow their blood (and therefore oxygen) supplies rapidly (Garcia-Bermudez et al., 2018). *In vitro* studies have shown restriction of the essential amino acid methionine slows GBM cell *in vitro* via inhibition of key oncologic signaling proteins including PI3K, p38MAPK, and ERK (Palanichamy et al., 2016; Palanichamy and Chakravarti, 2017).

Lastly, metabolism of the amino acids arginine and tryptophan have been linked to decreased detection by neighboring immune cells, which is crucial to successful tumor invasion (Kesarwani et al., 2018).

### Oxidative Phosphorylation and IDH Mutations

The tricarboxylic acid cycle (TCA cycle) is the central point of convergence for intermediates generated in the metabolic pathways altered in GBM (Strickland and Stoll, 2017). Carbohydrate, lipid, and amino acid metabolism generate metabolites that are fed into the TCA cycle in tumors through differential regulation of many of the pathways described above (Marie and Shinjo, 2011; Maus and Peters, 2017). Though the TCA cycle has at times been overlooked due to metabolic research in GBM being primarily focused on aerobic glycolysis, emerging evidence has indicated that GBM cells, especially certain cellular subtypes, utilize the TCA cycle to fuel energy production and biomolecule synthesis crucial for growth (Marin-Valencia et al., 2012; Shi et al., 2019).

Interestingly, it has been primarily genomic, rather than metabolic studies that have shed light on the importance of oxidative phosphorylation in GBM (Özcan and Çakır, 2016; Anderson et al., 2018). Several mutations in TCA Cycle enzymes and enzymes in adjacent metabolic pathways are commonly found in glioblastomas, and none more prevalently than isocitrate dehydrogenase 1 (IDH1) and 2 (IDH2) (Cohen et al., 2013; Quinones and Le, 2018).

IDH mutant tumors constitute roughly 10 percent of all glioblastomas (Cohen et al., 2013; Wu et al., 2019). Tumors with IDH mutations are typically secondary GBMs (Fack et al., 2015; Jahangiri et al., 2017; Chandra et al., 2020) and have a longer mean survival, and different mutation and histopathological profiles than their IDH wild-type counterparts (Cohen et al., 2013; Olar and Aldape, 2014). Additionally, IDH mutant tumors have metabolic profiles entirely distinct from IDH wildtype GBMs including an exaggerated dependance on glutamate as an energy source (Li et al., 2017; Maus and Peters, 2017). IDH1-mutatnt GBMs have a high demand for glutamate and are believed to use this amino acid as a chemotactic signal. As healthy astrocytes excrete glutamate, IDH1-mutated GBM cells tend to lack dense tumor structures, and instead migrate, invade, and disperse into adjacent cerebral tissue where glutamate concentrations are higher (Marin-Valencia et al., 2012; Maus and Peters, 2017). The abundance of IDH mutations in GBM, and the differences found in tumors with the mutation has led to IDH status being the primary mode of classification of GBM tumors (Cohen et al., 2013).

IDH1 and IDH2 are NADP^+^-dependent enzymes that interconvert isocitrate and α-ketoglutarate (αKG) in cytosol and mitochondria, respectively. Genome-wide exon-sequencing studies of gliomas have revealed IDH1 R132H activating point mutations in as many as 80–90% of low-grade gliomas (LGGs) (Cohen et al., 2013; Li et al., 2017). Mutations affecting IDH2 and additional IDH1 variants have also been reported in gliomas, but at a lower frequency, with the majority conferring similar changes in IDH activity (Cohen et al., 2013). The primary mechanism by which mutant IDH contributes to the pathogenesis of GBM is ascribed to the deregulated enzymatic activity of mutant IDH, which converts αKG into the metabolite D2HG, which in turn inhibits αKG-dependent dioxygenases, such as ten–eleven translocation (TET) family 5-methylcytosine DNA hydroxylases and the Jumonji C domain-containing histone–lysine demethylases (KDMs) (Xiao et al., 2012; Miller et al., 2017). Consequently, mutant IDH1and IDH2 activity cause aberrant DNA and histone methylation, which lead to widespread hypermethylation of cytosine–phosphate–guanine (CpG) islands a phenomenon termed the glioma CpG-island methylator phenotype (G-CIMP) (Malta et al., 2018).

**FIGURE 1:**
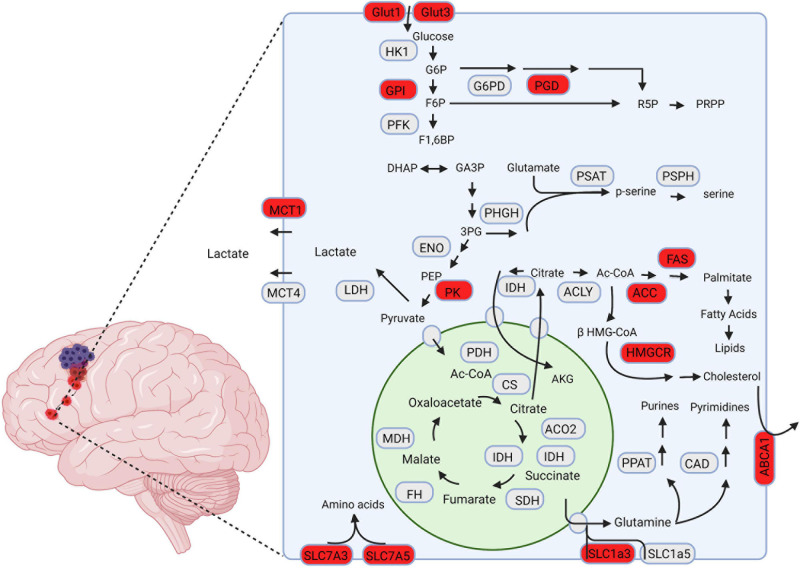
A schematic representation of metabolic pathways associated with GBM invasion. The proteins highlighted in red are hypothesized to drive invasion in GBM cells. Created using Biorender.com.

Mutations in additional TCA and TCA adjacent enzymes are found in GBM with lower frequency and comparatively fewer known functional implications compared to IDH (Cohen et al., 2013; Huang et al., 2019). Notably, mutation of fumarate hydratase (FH), and succinate dehydrogenase (SDH) have been reported and further solidify the TCA cycle’s importance in GBM’s metabolic reprogramming (Bardella et al., 2011; Schmidt et al., 2019). Further studies are needed to elucidate the advantages conferred to GBM cells through these mutations, and how they might contribute to GBM’s invasive phenotype. A graphical summary of the enzymes and pathways that have been tied to GBM tumor invasion is displayed in Figure 1.

## Effects of the GBM Microenvironment on Metabolism

### The Brain Extra Cellular Matrix as a Driver of Altered Metabolism

The brain extracellular matrix (ECM) occupies a notable portion of the CNS and contributes to its normal physiology (Goldbrunner et al., 1998). It is produced intracellularly and secreted to form a network of proteins and glycans, occupying the parenchyma of virtually all CNS cells (Bonneh-Barkay and Wiley, 2009; Lau et al., 2013). Structurally, the ECM provides cerebral cells with anchorage points and facilitates the organization of these cells into distinct CNS regions (Lau et al., 2013). It is a source of important molecular signals that influence cellular growth and survival (Bonneh-Barkay and Wiley, 2009).

Alterations to the ECM occur in many diseases of the CNS, including glioblastoma (Goldbrunner et al., 1998; Bonneh-Barkay and Wiley, 2009). As GBM cells navigate through the ECM, molecular changes allow the tumors to adhere to, detach from, and degrade ECM as needed to facilitate their invasive processes (Goldbrunner et al., 1998; Malric et al., 2017). Changes in cellular metabolism appear to be important players in many of the interactions between tumor cells and the ECM, though it is contested whether these changes are causative or merely consequential.

### Effects of GBM Cell Adhesion and Detachment on Tumor Metabolsim

During the invasive process, GBM cells become polarized, and their outer edge undergoes dynamic cytoskeletal rearrangements that facilitate adhesion to the adjacent ECM. This process is generally believed to be regulated by receptors in the integrin protein family (Malric et al., 2017). Integrins, which are characterized by a large extracellular domain, a short transmembrane domain, and a small intracellular non-catalytic cytoplasmic tail, are key components of the crosstalk between GBM cells and the microenvironment (Alberts et al., 2002; Bellail et al., 2004). Expression levels of several integrins are associated with poor prognosis and decreased survival in GBM patients (Paolillo et al., 2016; Malric et al., 2017).

Given the important link integrins provide between GBM cells and the tumor microenvironment, their role in tumor cell metabolism has been explored in several *in vitro* studies (Alberts et al., 2002; Paolillo et al., 2016). Unsurprisingly, a plethora of metabolism related signaling pathways have been shown to exert regulatory effects on integrins (Ata and Antonescu, 2017). Cellular signaling via the hypoxia related transcription factor HIF1α, mTOR signaling through amino acid relative abundance, and activity of the energy sensing protein AMP-Protein kinase have established regulatory effects on integrin function (Ata and Antonescu, 2017; Georgiadou et al., 2017; Ju et al., 2017). A better understanding of reciprocal integrin-metabolism interactions through targeted mechanistic studies is needed in the future.

In addition to their influence via integrins, several metabolites and metabolic pathways have direct effects on tumor cell adhesion properties. Upregulation of glycolysis and the PPP have both been associated with the functional activity of the adhesion proteins E-cadherin and P- cadherin (Liu et al., 2019; Sousa et al., 2019). Furthermore, increased levels of the metabolite Acetyl-coA directly promote cell-ECM adhesion by donating the necessary acetyl group for lysine acetylation in cross-linking (Lee et al., 2018). Specific metabolic dependencies such as these can potentially be exploited wither through small molecule inhibition or nutrient deprivation.

### Effects of GBM Proteases on Cellular Metabolism

While adhering to the ECM allows GBM cells to migrate and disperse throughout the cerebral microenvironment efficiently, invasive tumors must also slice through the matrix to seed tumor cells in adjacent brain tissue. This is primarily accomplished via specific degradation enzymes called proteases (Lakka et al., 2005; Paw et al., 2015). Links between proteases and the cellular metabolism of GBM have been poorly explored relative to other invasive factors (Strickland and Stoll, 2017). Existing studies have highlighted instances of both metabolic factors influencing protease activity and vice versa (Rao, 2003; Lakka et al., 2005). The role these enzymes play in GBM invasion and the paucity of data regarding the extent of their links to tumor metabolism represent an attractive target for research.

Matrix metalloproteinases (MMPs) are proteases believed to play a central role in GBM invasion, owed to their ability to degrade many brain ECM components (Hagemann et al., 2012). This family of zinc ion-dependent enzymes is broadly divided into six classes based on substrate specificity (Klein and Bischoff, 2011). Acting through both the degradation of the ECM and activation of pro-migratory signaling cascades, MMPs are able to promote highly invasive tumor behavior (Goldbrunner et al., 1998; Hagemann et al., 2012). The main MMPs implicated in GBM are the gelatinases MMP-2 and MMP-9, as well as the membrane-type MT1-MMP (MMP-14) (Hagemann et al., 2012; Ulasov et al., 2014; Zhang et al., 2019). Though MMPs are not typically viewed as important metabolic modulators, there is emerging evidence that MMP-2, MMP-9, and MMP-14 are potent modulators of cholesterol metabolism, a crucial pathway in glioblastoma growth and invasion (Ulasov et al., 2014; Hernandez-Anzaldo et al., 2016).

Urokinase-type plasminogen activator (uPA) is a serine protease which, along with its receptor uPAR, plays a role in invasion and neovascularization in gliomas (Lakka et al., 2001). Molecular characterization of high-grade gliomas has revealed increased expression of uPA and uPAR and has correlated upregulation of these genes with a more invasive tumor phenotype (Lakka et al., 2001; Chandrasekar et al., 2003). In addition to its intrinsic protease activity, uPA is able to indirectly activate other pro-form collagenases responsible for the degradation of plasmin-resistant ECM components (Zhou et al., 2000; Bekes et al., 2011). Upregulation of uPA has been shown to inhibit the Phosphoinositide 3-kinase (PI3K)–AKT signaling network which has diverse downstream effects on cellular metabolism, both through direct regulation of nutrient transporters and metabolic enzymes, and the control of transcription factors that regulate the expression of key components of metabolic pathways (Chandrasekar et al., 2003). The number of links found between tumor invasion and cellular metabolism in proteases in a relatively short amount of time warrant a deeper dive into this area of GBM research.

### GBM Stem Cells and Metabolism

Glioblastoma Stem Cells (CSCs) constitute a small percentage of GBM tumor cells and demonstrate two principal features of stem cells: self-renewal and differentiation (Lathia et al., 2015). GSCs are enriched in factors responsible for invasive potential and are found at the leading edge of recurrent tumors following surgical resection. These cells migrate along the vasculature and white matter tracts utilizing cadherins and integrins and cleave their way through extracellular matrix using matrix metalloproteinases such as MMP9 and ADAMT2 (Lathia et al., 2015; Prager et al., 2020). Additionally, several signaling pathways that are upregulated in GSCs, including L1CAM and ephrin-B2, have been shown to enhance tumor cell invasioveness. GSCs are non-autonomous cells and are crucial to the interplay between GBM tumors and their surrounding microenvironment (Prager et al., 2020). Thus, GSCs may represent an important link between GBM invasion and metabolism.

Distinct microenvironments in GBM tumors; including the hypoxic core, the perivascular niche, and the invasive tumor edge, add to the heterogeneity and the dynamic behavior of GSCs in these tumors (Lathia et al., 2015). GSCs hold the unique ability to rapidly adapt to the metabolic changes in various tumor microenvironments (Garnier et al., 2019). GSCs residing in the perivascular niche exhibit an entirely different metabolic profile than those residing in the hypoxic niche of the tumor core. GSCs in the perivascular niche show a proneural phenotype while the GSCs in the hypoxic core exhibit a mesenchymal phenotype (Garnier et al., 2019; Prager et al., 2020). While GBM cells predominantly express PKM2 which promotes aerobic glycolysis for their glucose metabolism, GSCs express both PKM2 and PKM1 (Prager et al., 2020). PKM1 promotes mitochondrial metabolism allowing GSCs to shift between aerobic glycolysis and oxidative phosphorylation. While GSCs in the perivascular niche rely mostly on glucose metabolism, glutamine dependency has been seen in the mesenchymal GSCs residing in hypoxic niche (Garnier et al., 2019).

These dynamic metabolic changes in GSCs create a challenge when selecting therapeutic targets, and warrants exploration of targeting multiple pathways simultaneously. For example, radiation targets highly proliferative cells sparing slow cycling cells (Lathia et al., 2015; Garnier et al., 2019). Therefore, a combination of radiation and metabolic inhibition may hold promise. Understanding the role GSCs play in tumor metabolism is likely a crucial step of solving the puzzle that is GBM.

### The Immune Systems and Metabolism in GBM Invasion

To invade adjacent brain tissue successfully, GBM cells must avoid detection and defend themselves from the host immune system. The immune composition of GBM’s microenvironment evolves with tumor stage but is predominantly comprised of immune suppressive cells (Pombo Antunes et al., 2020). There are distinct populations of immune-modulating macrophages, regulatory T cells (Tregs), T as well as dendritic cells (DC) present (Pombo Antunes et al., 2020). Recent studies have highlighted the intimate relationship between tumor metabolism and immune modulation, and the influence these processes have on tumor growth and progression (Chinnaiyan et al., 2012). Global metabolic profiling has identified distinct alterations that play key roles in immune modulation and the subsequent facilitation of tumor cell invasion (Chinnaiyan et al., 2012; Pearce and Pearce, 2013).

Following activation, T cells and DC undergo rapid expansion, with an accompanying increase in bioenergetic demand (Everts et al., 2012). These activated immune cells shift their metabolism by increasing glycolysis, increasing glucose uptake, decreasing carbon flux into the mitochondria, and enhancing lactate production (Ghesquière et al., 2014; Kesarwani et al., 2017).

This preference for glycolysis has also been demonstrated in other immune cells including macrophages, neutrophils, B cells and natural killer (NK) cells (Biswas, 2015). However, the elevated glycolysis exhibited by tumors cells leads to a microenvironment devoid of glucose which can significantly impact immune response (Singer et al., 2011; Becker et al., 2013). There is substantial evidence suggesting in the cerebral microenvironment, intense competition for nutrients exists between neural, immune, and tumor cells (Becker et al., 2013). Furthermore, accumulation of lactic acid due to enhanced glycolysis by tumor cells also impacts the immune cell function (Singer et al., 2011). Increased lactic acid can inhibit monocyte differentiation into DCs and increase transcription and secretion of pro-tumorigenic cytokines such as IL-23 and reduction of T-cell response (Correia et al., 2017). Therefore, enhanced GBM glycolysis has both active and passive inhibitory effects on immune cells in the adjacent microenvironment.

Glioma cells also influence their adjacent immune microenvironment through amino acid metabolic pathways via increased uptake of branched chain amino acids (BCAAs) (Silva et al., 2017). Glioblastoma cells overexpress branched chain amino acid transaminase 1 (BCAT1) which enhances excretion of branched chain ketoacids (BCKA) through MCT1 that influx into nearby macrophages and reduce their phagocytic ability (Silva et al., 2017).

Furthermore, GBM tumors promote macrophage and T cell dysfunction through expression of the ectonucleosides CD39 and CD73 (Xu et al., 2013; Yan et al., 2019). These integral membrane proteins induce the production of the immunosuppressive metabolite adenosine (Xu et al., 2013). Another link between amino acid metabolism and GBM immune invasion has been demonstrated in studies exploring the role of indoleamine 2,3-dioxygenase 1 (IDO1) (Zhai et al., 2015; Valtorta et al., 2020). IDO1 is an enzyme in the tryptophan metabolism pathway and converts tryptophan to kynurenine (Zhai et al., 2015). This conversion results in effector T cell energy, while concurrently promoting maturation and activation of Tregs blunting the host immune response to GBM cells (Munn et al., 2004). Pharmacologic inhibition of IDO has successfully enhanced the efficacy of immune checkpoint inhibitors in intracranial GBM mouse models (Wainwright et al., 2014).

Moreover, hypoxic conditions associated with the GBM tumor microenvironment can also contribute to immune system suppression (Wei et al., 2011). Hypoxia stimulates STAT3 phosphorylation which in turn activates immune-suppressive Tregs (Ben-Shoshan et al., 2008). Hypoxia also enhances the production of immune-suppressive cytokines including transforming growth factor (TGF)-β and vascular endothelial growth factor (VEGF) (Wei et al., 2011). Hypoxia also influences the immune cell composition of the adjacent microenvironment (Hussain et al., 2006; Wei et al., 2011). CNS macrophages are primary immune cells infiltrating GBM tumors and play an important role in mediating innate immunity in GBM (Pollard, 2004). These macrophages become tumor-associated macrophages (TAMs) when exposed to hypoxia (Lin et al., 2006). TAMs are polarized toward immune-suppressive and pro-tumorigenic phenotype (M2) via the STAT3 pathway (Pollard, 2004; Lin et al., 2006). In addition, TAMs further reinforce the GBM metabolic switch to aerobic glycolysis by secreting IL-6 which promotes the phosphorylation of phosphoglycerate kinase 1 (PGK1) and enhances its activity (Zhang et al., 2019).

In summary, tumor metabolism plays a crucial role in negating local immune detection and response. This dynamic interplay must be taken into consideration when targeting invasion in GBM.

## Opportunities to Target Metabolism in GBM as a Way of Targeting GBM Invasion

**FIGURE 2:**
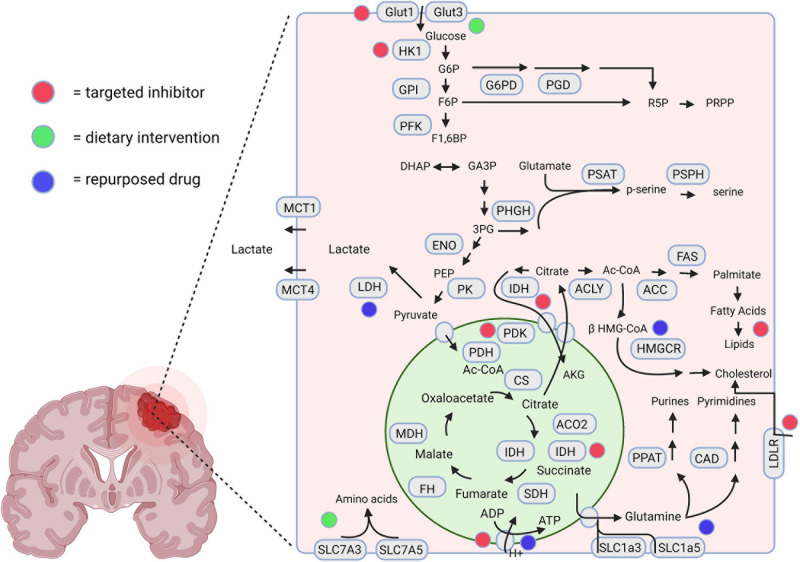
A schematic representation highlighting the therapeutic interventions associated with GBM metabolism. The red, green, and blue dots depict the interventions using novel targeted inhibitors, dietary interventions, and repurposed drugs, respectively. Created using Biorender.com.

The metabolic divergence between GBM cells and normal astrocytes holds potential for the discovery of novel therapeutic targets (Wolf et al., 2010; Nakano, 2014). Metabolomic and gene expression profiling can assist in the quantification of metabolites and metabolic enzymes in invasive cells, which can be combined to examine metabolic pathways of particular importance in these cells (Wolf et al., 2010; Chinnaiyan et al., 2012). Additionally, the tumor’s metabolomic profile can be correlated with the gene expression or proteomic profile of corresponding tissues, potentially yielding novel diagnostic or prognostic markers (Zhou and Wahl, 2019). Understanding how these metabolic networks vary and their importance for rapid invasion and proliferation may identify further targeted therapeutic strategies for GBM patients. A graphical representation of targeted metabolic inhibitors, dietary interventions, and repurposed drugs with potential utilization in invasive GBM is displayed in Figure 2.

### Metabolic Targets in GBM Resistant to Standard Therapies

Current standard treatment for GBM involves surgical resection followed by concurrent radio and chemotherapy (Wen and Kesari, 2008). Despite this aggressive treatment regimen, the median survival of GBM patients remains under 2 years (Davis, 2016). The devastating prognosis carried by this tumor is inseparable from its invasive capacity, which makes total surgical resection or treatment with local therapies extremely challenging (de Gooijer et al., 2018). When local therapies fail, systemic treatments are used to attempt to slow tumor growth. However, aspects unique to GBM, including shifts in its cellular metabolism, render these mainstay cancer treatments often ineffective, allowing the tumor cells to continue down the path of uncontrolled growth and invasion (Wolf et al., 2010; Zhou and Wahl, 2019).

Whole brain radiation has previously been shown to induce metabolic changes in GBM cells (Gupta et al., 2020). Untargeted metabolomics experiments have identified significant differences in metabolite levels pre and post radiation in both GBM and adjacent normal brain (Wibom et al., 2010; Gupta et al., 2020). Increased levels of glutamine and alanine and decreased levels of galactose and tyrosine were observed in biopsied tumor tissue post irradiation (Wibom et al., 2010). Additional pathway specific studies in GBM cell lines have shown that radiation induces a transition in metabolic preference from glycolysis to oxidative phosphorylation via mTOR mediated HEK2 inhibition (Lu et al., 2015; Zhou and Wahl, 2019).

Several studies have reported the metabolic changes associated with Temozolomide (TMZ) resistance. GBM treatment with TMZ induces changes in mitochondrial complex activity and glutamate metabolism (Oliva et al., 2010). TMZ resistant cells show increased levels of complexes II/III and CcO (complex IV) (Oliva et al., 2010; Ulasov et al., 2014). Selective inhibition of the OXPHOS components is currently being explored as a strategy to overcome this therapeutic resistance (Shi et al., 2019).

The VEGF inhibitor bevacizumab does not improve patient overall survival and has shown to be associated with increased invasion of GBM cells (Fack et al., 2015; Jahangiri et al., 2017; Chandra et al., 2020). VEGF inhibition results in a hypoxic tumor microenvironment which GBM overcomes through dynamic shifts in glucose uptake and carbohydrate metabolism (Kuang et al., 2017). Tumors treated with bevacizumab show enhanced glucose influx via upregulation of the glucose transporters GLUT1 and GLUT3, and an increased production of lactate compared to untreated cells (Fack et al., 2015; Kuang et al., 2017). While the correlation between these adaptive metabolic changes seen after bevacizumab treatment of GBM and the invasive phenotype seen in bevacizumab-resistant GBM has yet to be definitively established, the metabolic adaptations GBM cells undergo to overcome systemic cancer treatments represent an attractive target to unlock the full potential of anti-angiogenic therapy in GBM.

### Targeting Carbohydrate Metabolism

Among the metabolic pathways targeted in GBM by researchers, glycolysis has historically been the most prevalent (Wang et al., 2019). Most metabolic interventions to reach the clinical trial stage in GBM thus far have been dietary interventions rather than targeted inhibitors (Martin-McGill et al., 2017; Schwartz et al., 2018; van der Louw et al., 2019). Through the alteration of their intake composition, it is hypothesized that patients can deprive GBM tumors of the fuels that allow them to grow and proliferate (Schwartz et al., 2018). The ketogenic diet (KD), which aims to deprive GBM cells of their preferred metabolic fuel, glucose, aims to slow GBM growth and increase survival time among patients. The KD has been shown to reduce rodent tumor growth and tumor size and increase survival of animals (Lussier et al., 2016; Martin-McGill et al., 2018). Numerous clinical trials have tested different formulations of the ketogenic diet in GBM patients, and combined use of the KD with traditional chemotherapies (Martin-McGill et al., 2017; van der Louw et al., 2019). A summary of clinical trials using dietary modifications to treat GBM are summarized in Table 1.

**TABLE 1:** Clinical trials featuring dietary interventions to treat Glioblastoma multiforme.

**Dietary intervention**	**Author group**
Ketogenic Diet wherein ketosis is maintained by consuming a 60% medium chain triglyceride oil-based diet*	Nebeling et al., 1995
Restricted 4:1 (fat: carbohydrate + protein) ketogenic diet that delivered roughly 600 kcal/day	Zuccoli et al., 2010
Calorie Restricted Ketogenic Diet (CRKD) with fasting during and after concurrent radiotherapy	Rieger et al., 2014
Ketogenic Diet with < 30 g Carbohydrates Daily	Jameson, 2014
Ketogenic diet with concurrent radiotherapy and temozolomide chemotherapy	Woolf et al., 2016
Ketogenic diet using a 3:1 ratio of grams of fat to grams of protein + grams of carbohydrates	Klein et al., 2020
Modified Atkins Diet (MAD): 65% of total calories from fat, 25% from protein, and 10% from carbohydrates	Strowd et al., 2015
Modified Ketogenic Diet (MKD): 70% of total calories come from dietary fat, carbohydrate limited to 20 g/day	Martin-McGill et al., 2018
KD vs. control with intranasal administration of perillyll alcohol	Guimarães Santos et al., 2018

*Trial targeted malignant astrocytoma rather than glioblastoma.

The results of the aforementioned trials suggest the ketogenic diet can be safely implemented in patients and can achieve sufficient levels of ketogenesis (Santos et al., 2018; Schwartz et al., 2018). Patient recruitment and retention are two major factors which limit longer-term and larger trials (Martin-McGill et al., 2018; Schwartz et al., 2018; van der Louw et al., 2019). In those that have been conducted so far, a few smaller studies have shown modest effects on either tumor burden or progression (Martin-McGill et al., 2018). Optimization of timing and synergistic therapies are needed to assess whether the affects seen with this diet can match the results found across animal studies.

Carbohydrate metabolism has also been targeted in GBM both *in vitro* and *in vivo* with small molecules that disrupt key steps in crucial metabolic pathways. 2-deoxyglucose (2DG), which halts glycolysis following its initial phosphorylation by hexokinase, has been shown to inhibit GBM growth to a greater degree when combined with targeted radiation than radiation alone, and was safely used in a clinical trial in India with modest improvements in survival and reported patient quality of life (Dwarakanath et al., 2009; Shah et al., 2019). ManWZB117, a GLUT1 inhibitor, has been shown to inhibit tumor formation from GSCs but failed to limit progression of existing tumors (Shibuya et al., 2014). Additional GLUT1 inhibitors ritonavir and idinivar have been shown to reduce glucose consumption and GBM cell proliferation *in vitro*. 3-bromo-2-oxopropionate-1-propyl ester (3-BrOP), an inhibitor of hexokinase (HK) and 3-phospho dehydrogenase (3-PD) targets GSCs and therapeutic resistance acquired by GSCs. Combination treatment of 3-BrOP with carmustine has been shown to have a synergistic effect on decreased tumor formation (Shibuya et al., 2014). Dichloroacetate (DCA), a pyruvate dehydrogenase kinase (PDK) inhibitor has been shown to suppress tumor growth in GBM animal models, though its efficacy in human patients alone or in combination with other therapies remains to be seen (Yuan et al., 2013).

Finally, with advances in synthetic biology and biochemical techniques, molecular therapies focused on protein and gene level regulation are becoming the norm in cancer therapeutic research, and GBM is no exception. MicroRNAs (miRNAs) have come into focus as potential therapeutic targets to mediate the metabolic shifts in GBM due to their ability to regulate levels of specific metabolic genes of interest (Semonche et al., 2019). Examples include miR-106a which regulates GLUT3, miR-143 which regulates HKII, and miR-326 which regulates PKM2 (Dai et al., 2013; Zhao et al., 2013). Other investigators have attempted to inhibit tumor progression through modulation of miRNA-451 which exhibits negative regulatory effects on the LKB1/AMPK pathway (Godlewski et al., 2010). Exploitation of GBM’s downregulation of miRNA-451, which in turn results in increased cell migration through activation of the LKB1-AMPK pathway is currently being investigated with respect to its therapeutic potential (Godlewski et al., 2010; Ahir et al., 2017).

### Targeting Lipid Metabolism

Long overlooked in favor of other pathways, lipid metabolism has increasingly come into focus in the targeting of metabolic pathways in GBM. LXR-623 and archazolid B are two promising metabolic therapies and inhibit cholesterol uptake and recycling, respectively. One or both drugs can potentially be used to target cholesterol metabolism which has been linked to GBM’s invasive spread (Hamm et al., 2014; Villa et al., 2016). Arachidonyl trifluoromethyl ketone (AACOCF_3_) which inhibits cytoplasmic phospholipase A_2_ in phospholipid metabolism, and A922500, which inhibits diacylglycerol-acyltransferase 1 (DGAT1) in the formation of lipid droplets from FFAs, are two more targets that the inhibition of which has stunted tumor growth in intracranial xenograft mouse models (Anfuso et al., 2014; Cheng et al., 2020, p. 1).

### Targeting Oxidative Phosphorylation

Altering mitochondrial activity holds promise for targeting metabolism in GBM. Nigericin, a drug identified in a small molecule screen was able to induce mTOR inactivation, increase autophagy and decrease tumor growth *in vivo* (Hegazy et al., 2016). Metformin, a mainstay treatment for diabetes, has also shown potential in the treatment of GBM potentially through its effects on mitochondrial bioenergetics (Würth et al., 2013). Furthermore, treatment with Phenformin, an analog of Metformin, has been shown to decrease tumor growth and reduce markers of GSCs *in vivo*. Combination treatments using Phenformin and TMZ inhibit tumor growth *in vitro* and *in vivo* (Jiang et al., 2016). An arsenic based mitochondrial toxin, 4-(N-(S-penicillaminylacetyl)amion) phenylarsonous acid (PENAO) in combination with DCA results in inhibition of GBM proliferation, and induces G2/M cell cycle arrest both *in vitro* and *in vivo* (Shen et al., 2015). Recently, researchers identified the compound benzimidazolinium Gboxin. This small molecule, which inhibits ATP synthase activity in a proton gradient dependent manner, is a novel inhibitor of oxidative phosphorylation, and targets the specific mitochondrial alterations found in cancer cells (Shi et al., 2019). Further studies confirmed Gboxin accumulation and growth inhibition in GBM allografts and patient derived xenografts, providing additional validation of OXPHOS as a viable therapeutic target in GBM.

While therapies targeting metabolic pathways to reduce invasion in GBM have shown tremendous promise, there are multifactorial challenges in utilizing these treatments in patients. The suppression of invasion via inhibition of cellular metabolism in GBM is subject to the same difficulties faced by pharmacologic treatments of any disease of the central nervous system, that is, penetration of the BBB (Daneman and Prat, 2015; Strickland and Stoll, 2017). Furthermore, the incredible heterogeneity of GBM’s molecular profile pose the same challenges in choosing specific targets of treating any malignant neoplasm (Hatoum et al., 2019). Finally, therapies focused on metabolic pathways meet the new dilemma of targeting factors that drive the metabolism of normal and cancerous cells alike (Strickland and Stoll, 2017). Successful future therapies will not only have to suppress the metabolism that drives invasion in tumors but do so in a way that non-cancerous cells are not significantly harmed.

### Drug Repurposing

Although potential therapies targeting tumor metabolism hold promise in the treatment of invasive GBM, several challenges in the drug development pipeline exist (Seliger and Hau, 2018; Tran and Prasad, 2020). Drugs designed for diseases other than cancer, such as antibiotics and antidiabetic agents, have the potential to be used as anti-tumor agents. Drug repurposing is best defined as the utilization of approved drugs, outside the scope of their original indication (Tran and Prasad, 2020). The advantage of repurposed drugs is that they circumvent many of the laborious and time-consuming challenges in the drug development pipeline, making them an attractive strategy in cancer therapeutic research (Seliger and Hau, 2018). Many pharmaceuticals with metabolic targets exist as strategies to treat other diseases, including heart disease and diabetes. Several of these drugs are currently being researched in animal models and in clinical trials as a method of slowing cancer growth, with GBM being no exception (Jiang et al., 2016; Seliger and Hau, 2018).

Metformin, an example of a repurposed drug with growing interest for its potential to target tumor metabolism, is a first-line treatment for type II diabetes (Kasznicki et al., 2014). Metformin has been reported to possess anticancer properties affecting the survival of cancer stem cells in breast cancer models, likely through the regulation of AMP kinase (AMPk), triggered by reduction in ATP/AMP ratio (Würth et al., 2013; Kasznicki et al., 2014). Treatment with metformin reduced the proliferation rate of tumor-initiating cell-enriched cultures isolated from four human glioblastomas. Metformin has also been shown to impair tumor-initiating cell spherogenesis, indicating a direct effect on self-renewal mechanisms (Würth et al., 2013). Additional studies in cell models have shown metformin can inhibit migration of GBM cells.

Another commonly used drug group, statins, have been implicated for potential repurposing to treat glioma based on promising *in vitro* studies and observational research in patient cohorts (Fatehi Hassanabad, 2019). Statins inhibit 3-hydroxy-3-methylglutaryl-CoA reductase, the rate-limiting enzyme of the mevalonate pathway. It has been proposed that statins induce tumor-specific apoptosis through mitochondrial apoptotic signaling pathways, which are activated by the suppression of mevalonate or geranylgeranyl pyrophosphate biosynthesis (Gong et al., 2019). These drugs have shown the ability to limit migration in cultured tumor cells, which some researchers have speculated could translate to slowing tumor invasion *in vitro* (Bababeygy et al., 2009). Existing evidence in humans has been less straightforward. A Meta-analysis showed no progression-free survival benefit or overall survival benefit to those patients taking statins (Fatehi Hassanabad, 2019).

Additional drugs that have been repurposed to stymie invasion in GBM include, NSAIDS, Disulfiram, and Ritonvir. These act through COX-2 inhibition and downstream reduction of c-myc and LDH expression, inhibition if ALDH and oxphos, and disruption of glutamine metabolism, respectively (Seliger and Hau, 2018). The evidence for these drugs benefitting GBM patients comes, again, from primarily basic science work in cell culture models (Maiti, 2014; Xu et al., 2015). Retrospective survival analyses on patients taking these drugs has shown mixed results, with modest survival benefits observed in some cohorts and no effect at all observed in others (Agarwal et al., 2017; Seliger and Hau, 2018). In addition to being largely inconclusive, such studies are commonly criticized for selection bias and immortal-time bias (Jiang et al., 2016; Tran and Prasad, 2020). Future well-designed, multicenter randomized controlled clinical trials will likely be needed to gauge the potential therapeutic benefit of these drugs.

## Conclusion

The collective body of knowledge concerning the metabolic profile of invasive glioblastoma is rapidly evolving. The study of tumor metabolism is shifting from a static picture shaped by genetic mutations or environment alone to a dynamic view in which genotype and microenvironment interact to form the metabolic profile of invasive tumor cells, exposing potential vulnerabilities that are ripe for therapeutic exploitation. Research in this area is challenging because it is not amenable tracking data at any particular point in time, but rather requires a nuanced understanding of physiologic and biochemical alterations in flux, especially in the dynamic environments which brain tumors encounter. Fortunately, novel clinical and research tools are providing powerful insights. By integrating the full spectrum of system-wide, unbiased “omics” screens available to researchers, and cutting edge diagnostic clinical tools including radiotracer and flux imaging technologies, a new understanding of the molecular basis of glioblastoma metabolism has begun to emerge, including metabolic vulnerabilities that could be targeted therapeutically. Additionally, the development of integrative models based on tumor molecular markers, microenvironment, or both are continuously revealing additional therapeutic targets. This review has focused primarily on the actionable targets and routes of exploration that will be the most useful for finding them, with the goal of expanding the breadth of pharmacologic interventions through new drug development and repurposing. Glioblastoma research has been at the forefront of cancer research for the past 10 years, including the elucidation of critical mechanisms by which tumors undergo metabolic reprogramming. It is time for this research to translate to the clinical setting for the benefit of patients.

## Author Contributions

JG prepared the figures and table. All authors contributed to the conception, drafting, and revising of the manuscript.

## Conflict of Interest

The authors declare that the research was conducted in the absence of any commercial or financial relationships that could be construed as a potential conflict of interest.
